# Copolyester toughened poly(lactic acid) biodegradable material prepared by *in situ* formation of polyethylene glycol and citric acid

**DOI:** 10.1039/d4ra00757c

**Published:** 2024-04-05

**Authors:** Xipo Zhao, Peidong Li, Fan Mo, Yuejun Zhang, Zepeng Huang, Jiajie Yu, Ling Zhou, Siwen Bi, Shaoxian Peng

**Affiliations:** a Hubei Provincial Key Laboratory of Green Materials for Light Industry, New Materials and Green Manufacturing Talent Introduction and Innovation Demonstration Base, Hubei University of Technology Wuhan 430068 China xpzhao123@163.com

## Abstract

Polylactic acid (PLA) is a high-modulus, high-strength bio-based thermoplastic polyester with good biodegradability, which is currently a promising environmentally friendly material. However, its inherent brittleness has hindered its widespread use. In this study, we reported a simple and non-toxic strategy for toughening PLA, using biodegradable materials such as polyethylene glycol (PEG) and citric acid (CA) as precursors. Through reactive melt blending with PLA, PEG and CA form PEGCA copolyesters *in situ* during blending. At the same time, CA can react with PLA and PEG, forming a copolyester structure at the interface of the two phases, improving the interfacial compatibility between PEG and PEGCA with PLA. Fourier transform infrared spectroscopy confirms this. Experimental results show that when the content of PEG/CA reaches 15% (PLA/PEG/CA-15%) in the blends, the impact strength of the blend was 4.47 kJ m^−2^, and the maximum elongation at break was as high as 360.1%, which were about 2 and 44 times higher than those of pure PLA, respectively. Moreover, the tensile strength was still maintained at the level of 70%. This work can expand the application of PLA in food packaging and medical supplies.

## Abstract 2

A simple and non-toxic strategy for toughening PLA.

## Graphical abstract

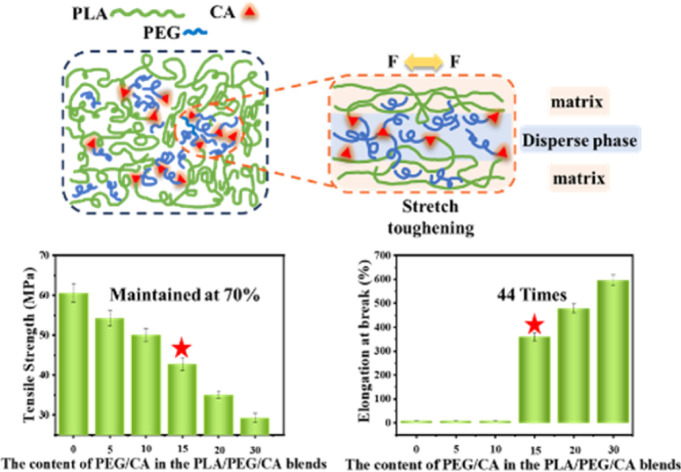

## 1. Introduction

With the dependence of many polymer materials on fossil resources, the scarcity of crude oil supply, and severe environmental pollution, the development of sustainable green polymer materials has become a hot topic in material science research.^1–4^ Polylactic acid (PLA), a biodegradable thermoplastic polymer from plants, can be decomposed into water and carbon dioxide in the natural environment and also be converted into raw materials through the photosynthesis, making it free from dependence on non-renewable resources such as oil. The excellent mechanical properties of PLA, particularly tensile modulus and strength, have attracted interest in fields such as packaging materials,^5^ automotive interiors and exteriors,^6^ and agriculture.^7^ Moreover, the good biocompatibility of PLA makes it an attractive polymer in biomedical applications, indicating its great application potential in medical materials such as tissue materials^8^ and drug sustained-release carriers.^9^

However, the inherent brittleness of PLA limits its application in many fields.^10^ In research, traditional methods such as copolymerization, blending,^11^ and plasticization^12^ are often used to modify PLA. Although chemical copolymerization can effectively improve the toughness of PLA, it often leads to serious loss of other properties of PLA. In addition, the high cost caused by long reaction cycles and harsh copolymerization conditions is another challenge for chemical copolymerization.^13^ By contrast, physical blending with plasticizers or flexible polymers is a simple, efficient, and cost-effective way to improve the toughness of PLA. At present, polyethylene glycol (PEG),^14,15^ propylene glycol,^16,17^ epoxy soybean oil,^18^ citric acid (CA) ester,^19,20^ poly butyl acrylate,^21,22^ and phthalate^23^ are functional small-molecule plasticizers. PEG, as an excellent green plasticizer, can remarkably change the brittleness of PLA and improve its toughness.^24,25^ However, similar to other plasticizers, PEG has easy migration and poor compatibility, making it difficult to maintain high toughness and mechanical strength, simultaneously.^26^ In improving compatibility, chemical grafting or adding chain extenders is an effective method. By melt reactive blending with PLA, the interfacial adhesion and compatibility between PLA and plasticizer are improved, thereby increasing tensile and impact toughness. Choi^27^*et al.* connected PEG with double bonds at one end of the PLA molecular chain through chemical grafting, reducing PEG leakage from PLA. When the PEG content of the blends was 20 wt%, the elongation at break was higher than 220%, and the impact strength can reach up to 62 kJ m^−2^. Liu G *et al.*^28^ prepared PLA/PEG blends *in situ* with the phenylmethane diisocyanate (PMDI) as chain extender. The hydroxyl groups at the end of PLA and PEG molecules reacted with the isocyanate group of PMDI to form PLA toughened by cross-linked polyurethane and the elongation at the break of the blend was increased to 200%.

Branched polymers are widely used for toughening polyester because of their low melt viscosity, high-end functional groups, and easy entanglement of long branched chains.^29,30^ Yang^31^*et al.* toughened PLA with a series of branched polycaprolactone (BPCL) with different chain lengths. They found that a longer CL chain length increased the mutual entanglement in the blends, reduced the hydrogen bond between BPCL_*x*_ and PLA, and increased the entanglement of the BPCL_*x*_ chain. The impact strength of the PLA/BPCL blend reached 23.34 J m^−1^, and the elongation at break was 6.58%. Bhardwaj ^32^*et al.* developed a nanocomposite of poly(lactide) (PLA) with excellent stiffness toughness balance by *in situ* crosslinking of hyperbranched polymers with hydroxyl functional groups and polyanhydrides in the PLA matrix, with the toughness of up to 17.4 MJ m^−3^ and elongation at break of 48.3%. Although these tasks can achieve good toughness, they often require pre-polymerization, reblending, or branched precursors in the early stage, which have a complex process and high cost.

In this work, PLA toughened by a PEGCA polyester was formed *in situ* by melt blending of bifunctional (–OH) PEG and trifunctional (–COOH) CA. The effects of different PEG/CA contents on the properties of PLA were investigated from tests on mechanical properties, thermal stability, and phase morphology, and the interfacial compatibility of the dispersed phase with the matrix as well as the mechanism of toughening PLA with PEGCA were discussed.

## 2. Experimental

### 2.1. Materials

PEG (*M*_w_ = 4000 g mol^−1^) and CA were obtained from Sinopharm Chemical Reagent Co., Ltd, China. PLA (2002D, density = 1.24 g cm^−3^) was purchased from NatureWorks LLC (USA).

### 2.2. Preparation of PLA/PEG/CA blends

PLA was dried in a vacuum at 60 °C for 12 h before blending. PLA and PEG were blended in accordance with the mass ratio of 100/0, 95/5, 90/10, 85/15, and 80/20. The total amount of feed was set as 50 g, in which the additional amount of CA is calculated in accordance with the molar ratio of functional groups of –COOH of CA and –OH of PEG as 1 : 1. PLA, PEG, and CA were mixed at 180 °C for 13 min in accordance with the ratio using the Harper torque Rheometer (RM-200), and the rotor speed was 60 rpm. All samples were hot pressed into 1 and 4 mm thin sheets at a pressure of 10 MPa using a 180 °C hot press machine, then cold pressed and shaped, and cut into tensile and impact test strips in accordance with the national standards using a sample cutter. The sample was labeled as PLA/PEG/CA-5%, PLA/PEG/CA-10%, PLA/PEG/CA-15%, and PLA/PEG/CA-20% in accordance with the ratio. For example, in PLA/PEG/CA-10%, 10% represents a PLA/PEG mass ratio of 90/10 in the blend, and the molar ratio of CA to PEG functional groups is 1 : 1.

### 2.3. Characterization and material property testing

#### 2.3.1. Fourier transform infrared spectroscopy

Fourier transform infrared spectroscopy (FTIR) (Iso10, Nicolet, USA) was used to study the reactions of PEG, CA, and PLA during blending at a resolution of 4 cm^−1^ and a scanning frequency of 32.

#### 2.3.2. Gel content measurement

The samples were dried in a vacuum oven at 60 °C for 8 h, then weighed by mass *W*_1_ and immersed in dichloromethane, where the PLA and cross-linked fraction are insoluble in dichloromethane. Thus, the crosslinked fraction can be separated from the solution by ultracentrifugation at 10 000 rpm for 15 min. The resulting product was dried in a vacuum oven at 40 °C for 24 h and weighed (*W*_2_). The gel content (*G*) can be calculated by using the following equation.
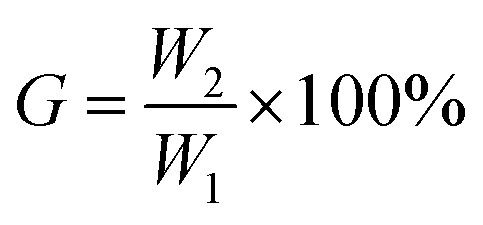
where *W*_1_ is the mass of the sample before extraction and *W*_2_ is the total amount of gel after extraction.

#### 2.3.3. Determined by gel permeation chromatography (GPC) measurement

Gel permeation chromatography (GPC, PL50, Agilent, USA) was used for determination, tetrahydrofuran was used as eluent (1 ml min^−1^), good solubility was maintained at 30, and polystyrene (PS) standard was used for calibration.

#### 2.3.4. Dynamic mechanical analysis

A single cantilever mode equipped with a dynamic mechanical analyzer (DMA Q800, TA, USA) was used to study the dynamic thermo-mechanical properties of PLA/PEG/CA composite materials. The test temperature was increased from −70 to 80 °C at a rate of 3 °C min^−1^, and the frequency was always maintained at 1 Hz.

#### 2.3.5. Differential scanning calorimetry (DSC)

Differential scanning calorimetry (DSC) was performed using a PerkinElmer DSC instrument (model 8000, USA) equipped with a liquid nitrogen cooling accessory. The samples were heated up to 200 °C for 5 min to eliminate thermal history, then cooled down to 0 °C at a rate of 10 °C min^−1^, and then heated up to 200 °C at a rate of 10 °C min^−1^. The crystallinity of PLA (*X*_c_) was estimated using the following equation.
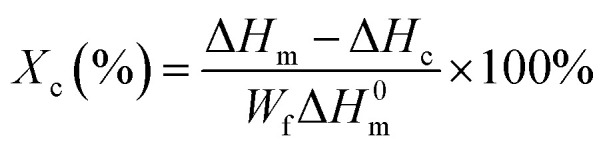
where Δ*H*_m_ and Δ*H*_c_ are the enthalpies of melting and cold crystallization during heating, respectively; Δ*H*^0^_m_ is the enthalpy assuming 100% crystalline PLA homopolymers (93.7 J g^−1^), and *W*_f_ is the weight fraction of the PLA component in the blends.

#### 2.3.6. Rheological behavior analysis

The prepared hot-pressed 1 mm thick samples were cut into a disk with a diameter of 25 mm, and the linear rheological behavior of the blend was obtained by rotating the plate rheometer (AR2000EX, TA, USA) in the plate–plate geometry at 180 °C. The frequency ranged from 0.1 Hz to 100 Hz, and the strain was 0.1%.

#### 2.3.7. Mechanical properties measurements

The hot-pressed 1 mm thick samples were cut to obtain a dumbbell-shaped sample in accordance with the international standard ISO 527–2, and the tensile rate of 10 mm min^−1^ on a CMT-4204 electric tensile tester (Shenzhen SANS, China) was used to obtain the stress–strain curve and tensile strength and elongation at break of the sample. The 4 mm thick samples were cut to obtain a sample in accordance with the international standard ISO 179–1, and the impact energy was measured by Charpy impact testing (SJJ-50, Chengde Jinjian, China) with a range of 1 J. In addition, the impact strength of the sample was obtained after calculation. All samples were prepared at least 5 or more times to obtain an accurate average value.

#### 2.3.8. Scanning electron microscopy

The morphologies of cryo-fractured and impact-fractured surfaces were examined by scanning electron microscopy (SEM, JSM-6390LV, JEOR, Japan) at an accelerating voltage of 10 kV. The 4 mm-thick samples were cryo-fractured and cut quickly after 30 minutes in liquid nitrogen.

## 3. Results and discussion

### 3.1. Structural analysis of blends

**Fig. 1:**
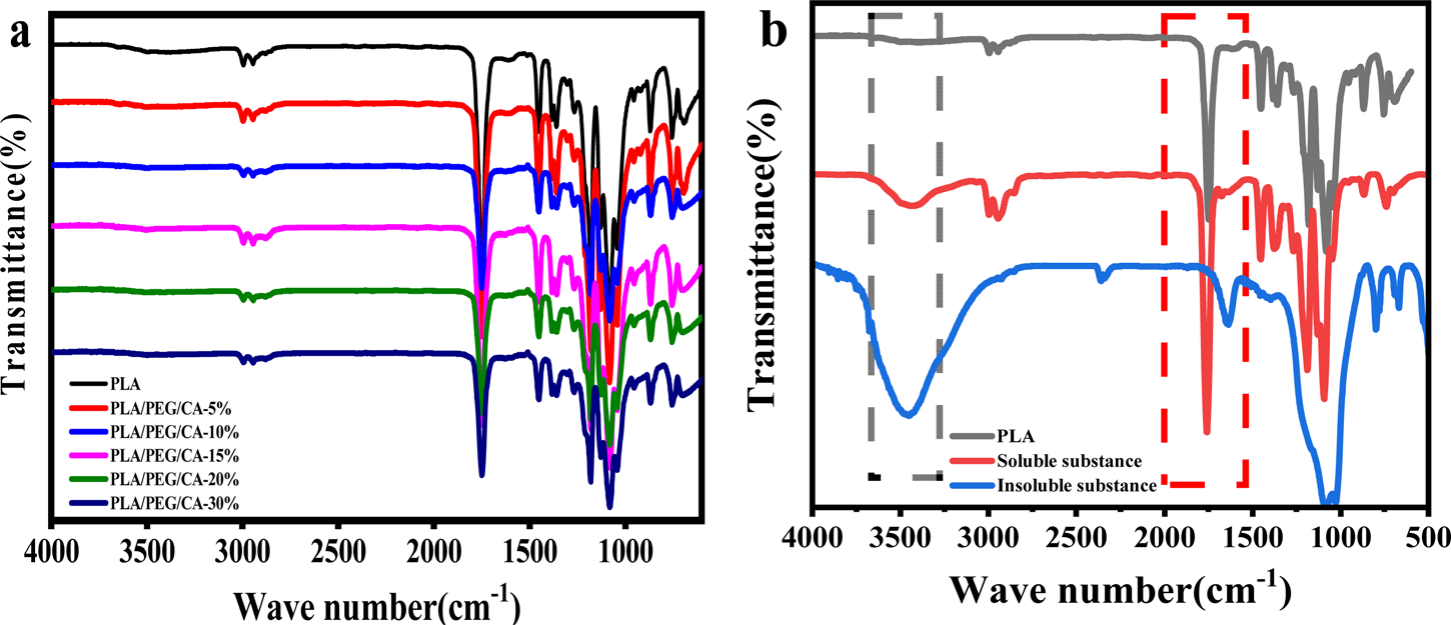
FTIR spectra of neat PLA and PLA/PEG/CA composite.

In this work, we aim to improve the toughness of PLA by producing copolyester PEGCA by *in situ* blending of CA and PEG. In addition, CA, as a compatibilizer and chain extender, uses its multiple carboxyl groups to react with the terminal hydroxyl groups of PLA and PEG, resulting in the formation of a copolyester structure at the interface between the matrix and the dispersed phase, thereby improving the interface compatibility with PLA.^33^ During the blending, the mobility of macromolecular segment PLA is low, and CA, as a small molecule, preferentially reacts with short-chain PEG to produce PEGCA copolyesters. The control of the molar ratio of hydroxyl groups of polyethylene glycol and carboxyl groups of citric acid is to reduce the production of PEGCA crosslinked products in the system. Fig. 1(a) shows the infrared spectra of PLA/PEG/CA blends with different contents. The results showed that the blends exhibited infrared characteristic peaks of PLA. Due to the high content of PLA, maybe it was difficult to see the characteristic peaks of a small amount of PEG and CA in the blends. To further illustrate the reaction of the blend system, the PLA/PEG/CA blends were dissolved in acetone, and the insoluble gel and solution were separated. The solution was added to a large amount of ethanol for precipitation and purification. The supernatant (the unreacted PEG, CA, and the non-cross-linked PEGCA) was separated to obtain polymer precipitation. The crosslinked insoluble matter and the purified dissolved product were analyzed by infrared spectroscopy. Compared with pure PLA, the dissolved and purified part showed a wider hydroxyl peak at 3497 cm^−1^, and the stretching vibration peak of C=O at 1759.3 cm^−1^ increased, indicating that more hydroxyl and ester groups were produced in the product after the blending reaction. It was speculated that the dissolved parts may be PLA and copolymer produced by the reaction of PLA and PEGCA. The insoluble part showed a wider hydroxyl peak and the C–O peak of ester. The group disappeared at 1234.1 cm^−1^, showing the C–O stretching vibration peak of ether bond at 1075 cm^−1^, and the stretching vibration peak of 1759 cm^−1^ C=O shifted, indicating that it may be mainly the crosslink of PEGCA.

To further explain whether PEGCA reacted with PLA *in situ* to form copolyesters during the blending process, the molecular weight of the blend after acetone dissolution and purification was tested as shown in Table 1. The results showed that compared with the molecular weight and molecular weight distribution of PLA treated in the same way, the weight average molecular weight and molecular weight distribution PDI of the product were increased after adding PEG/CA, which confirmed the speculation of the above infrared spectrum results.

**Table 1:** Molecular weight and distribution of blends after dissolution and purification

Sample	*M*_w_ (10^5^ g mol^−1^)	*M*_n_(10^5^ g mol^−1^)	PDI
Neat PLA	4.48	3.56	1.26
Soluble substance	4.69	3.31	1.42

**Fig. 2:**
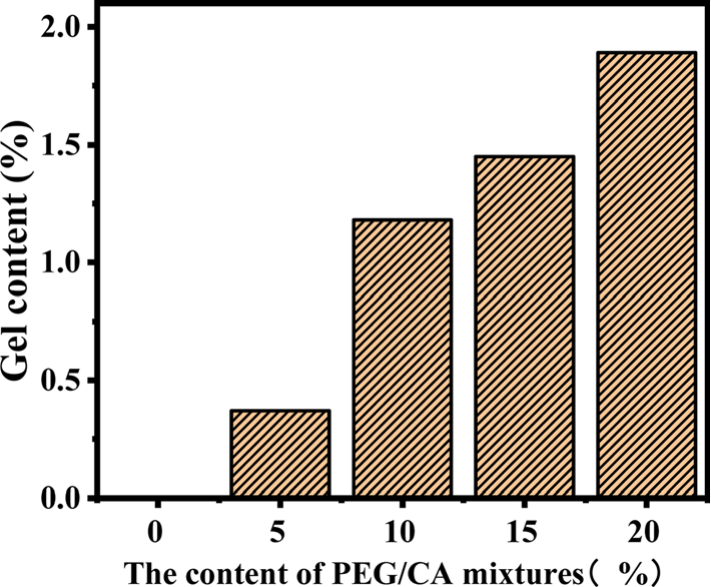
Gel content data of PLA blends with different PEG/CA content.

The gel content of the blend samples with different PEG/CA contents was tested, and the results are shown in Fig. 2. The gel content in the system was less, which indicates that the addition of PEG and CA with the molar ratio of functional groups of 1 : 1 can reduce the production of PEGCA crosslinks. With the increase of PEG/CA content, the increase of gel content in the blend was small, which also showed that the reaction activity of CA was low, and it was difficult to produce an ideal cross-linking structure with macromolecular chain PLA.

### 3.2. Torque analysis

**Fig. 3:**
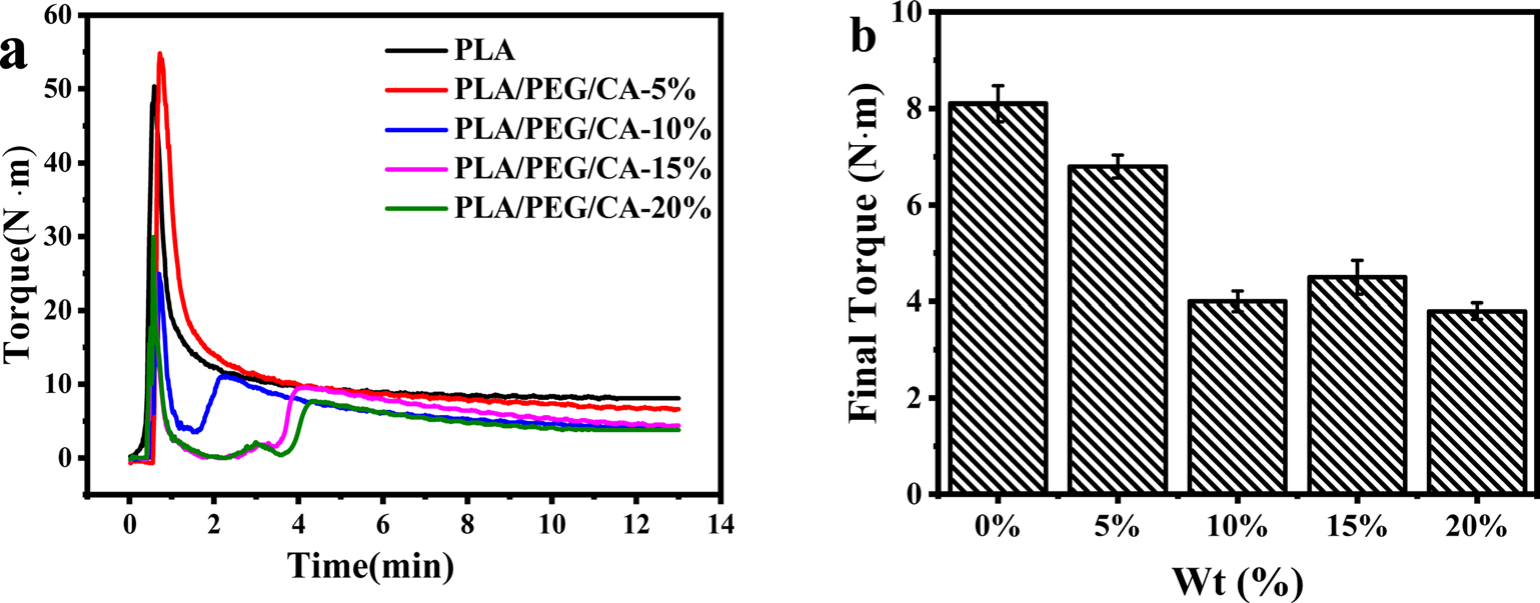
Variations of the reaction time on torque (a) and final torque (b) for PLA/PEGCA composites.

In the blending process, the variation of torque in the torque rheometer can reflect the reaction process and degree of the blending system. Fig. 3(a) shows the variation of the transition moment of the melt overtime during the blending process at 180 °C. The addition of small molecular PEG/CA components reduced the PLA feeding torque. With the extension of blending time, the torque entered a stable platform and the reaction tended to be complete. Fig. 3(b) shows that after adding PEG/CA, the final torque of the reaction first decreases and then slightly increases. The results showed that at 180 °C, the low viscosity PEG/CA tended to separate into the high shear region of the blend cavity, which mainly played the role of plasticization, and then the melt viscosity of the blend system decreased, and the equilibrium torque value at the end of the reaction decreased.^34^ Interestingly, when the PEG/CA ratio is 15%, the final torque increases slightly, which may be due to the increase of PEGCA *in situ* reaction, which increases the possibility of copolyester reaction with PLA and improves the chain entanglement with PLA.

### 3.3. Dynamic thermo-mechanical properties of PLA/PEG/CA blends

**Fig. 4:**
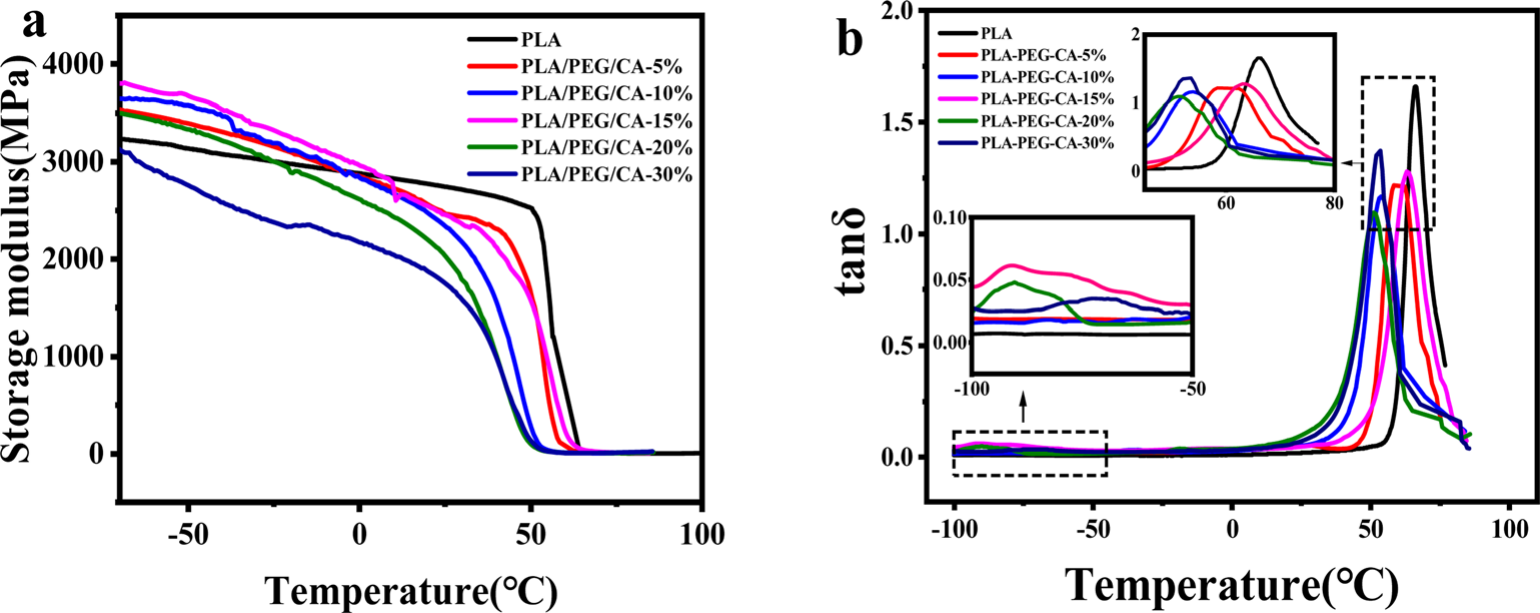
DMA traces of neat PLA and PLA/PEG/CA composites storage (a) modulus and tan *δ* (b) *versus* temperature.

Dynamic thermodynamic analysis (DMA) was used to analyze the phase separation and compatibility of the blends. In Fig. 4(a), it can be seen from the change of storage modulus of pure PLA and blending temperature that there is only one sudden drop of storage modulus, which indicates that there is no obvious phase separation in the blend. The storage modulus curve of pure PLA decreases sharply to near 60 °C, which corresponds to the glass transition region of pure PLA. With the increase of PEG/CA content, it moved to the low-temperature region, indicating that PEGCA formed *in situ* and played a certain plasticizing role. In Fig. 4(b), for the PLA/PEG/CA blend system, there are two peaks on the tan *δ versus* temperature curve of the blends, where the peak around 65 °C corresponds to the *T*_g_ of the PLA matrix, and the peak below 0 °C corresponds to the *T*_g_ of the branched component of the blend that forms PEGCA *in situ*, and the *T*_g_ values of the two components obtained from the tan *δ*–temperature curves are summarized in Table 2.

**Table 2:** *T*_g_ of neat PLA and different components of PLA/PEG/CA blends

Temperature (°C)	*T*_g(PLA)_ (°C)	*T*_g(PEGCA)_ (°C)
Neat PLA	66.0	–
PLA/PEG/CA-5%	58.4	26.8
PLA/PEG/CA-10%	54.2	−31.5
PLA/PEG/CA-15%	63.0	−91.5
PLA/PEG/CA-20%	54.5	−73.7
PLA/PEG/CA-30%	53.9	−77.2

As shown in Table 2, the relaxation peak corresponding to the PLA matrix and PEGCA components also varies with the PEG/CA content. With the increase of PEG/CA component content, the *T*_g_ value of PEGCA shifts to low temperature, which may be due to the formation of partially branched PEGCA due to the uneven diffusion of CA, and the flexibility of its molecular chain increases. The addition of PEG/CA reduced the intermolecular force of PLA and the *T*_g_ of PLA decreased. When the PEG/CA content was 15%, the *T*_g_ mutation of the PLA matrix increased, which may be due to the increase in the number of branched PEGCA in the system and the formation of copolyesters with macromolecular PLA, which increased the degree of entanglement between molecular chains in the blend system and hindered the movement of chain segments. This result also corresponded to the increase of final torque in DMA. However, with the continuous increase of PEG/CA content, the *T*_g_ of PLA matrix decreased, which may be due to the limited reaction between PEGCA and macromolecular segment PLA and the failure to produce more copolyester structure. Therefore, PEGCA mainly played a plasticizing role and the segment mobility increased.

### 3.4. DSC

**Fig. 5:**
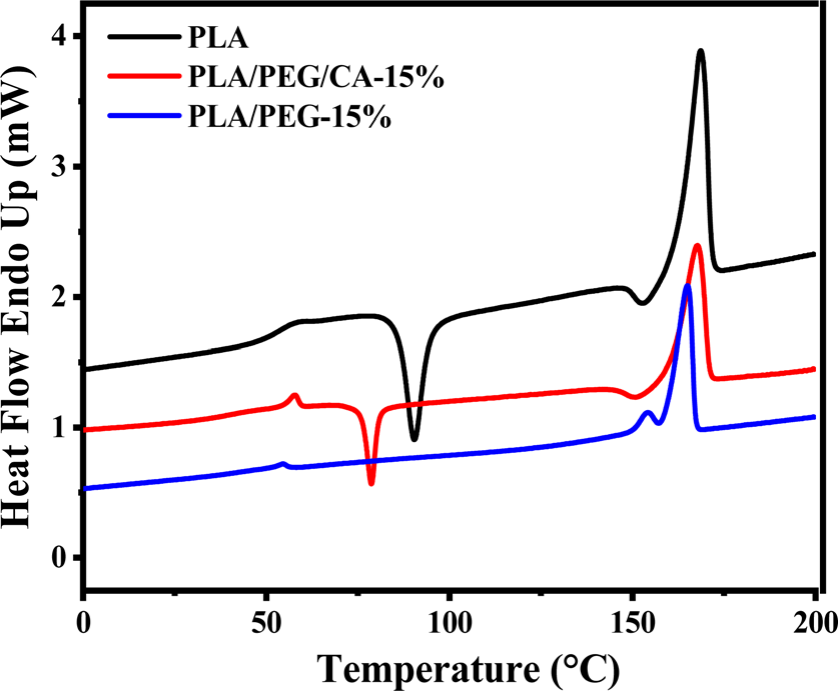
DSC curves of neat PLA, PLA/PEG, and PLA/PEG/CA blends.

Fig. 5 shows the second heating DSC curve of PLA/PEG blend after the addition of CA. The *T*_g_ of pure PLA is 60.56 °C, and the addition of plasticizer PEG results in a decrease in the *T*_g_ of the PLA matrix in the blend. This indicates that PEG weakens the intermolecular forces between the molecular chains of PLA, effectively increases the mobility of molecular chain segments, and leads to a decrease in *T*_g_ of the PLA matrix. At the same time, the cold crystallization peak of the PLA/PEG blend disappeared, indicating that the addition of PEG improved the crystallization ability of the PLA matrix. When the CA was added, the cold crystallization peak appeared instead, and *T*_g_ increased slightly, which further indicated that PEGCA and PLA reacted *in situ* to form copolymer, which increased the chain entanglement between PEG dispersed phase and PLA matrix, reduced the mobility of PLA chain segment, and was not conducive to crystallization. However, compared with pure PLA, the cold crystallization peak moved to a low temperature, indicating that it still played a plasticizing role as a whole.

**Fig. 6:**
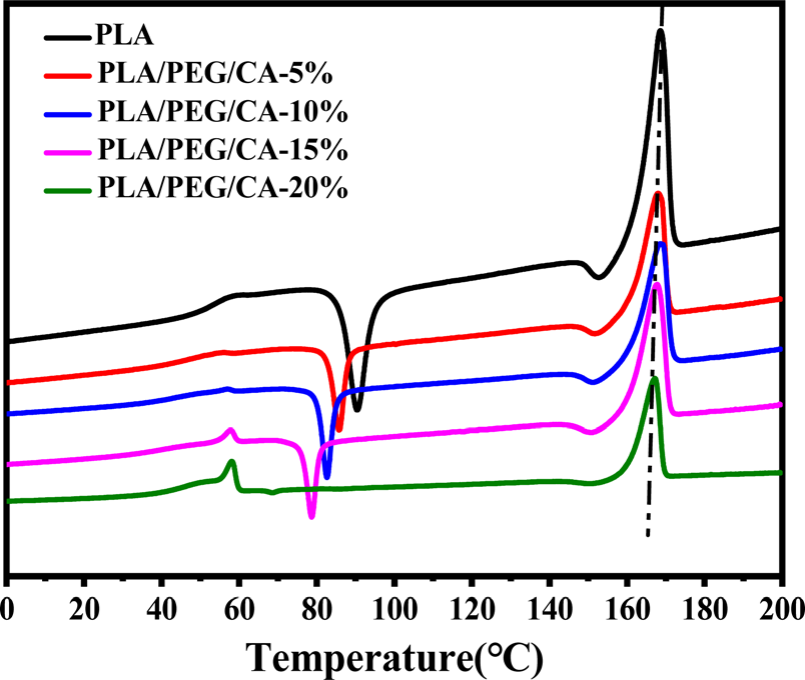
DSC heat curves of neat PLA and PLA/PEGCA composites.

Fig. 6 shows the DSC reheating curves of PLA blends with different PEG/CA contents, and Table 3 summarizes the data of DSC analysis. As shown in the table, the *T*_g_ value of the blends is lower than that of pure PLA, and the melting enthalpy (Δ*H*_m_) and crystallinity (*X*_c_, PLA) were higher than those of pure PLA. This result is mainly due to the introduction of the PEG/CA component, which enhances the plasticizing effect and the movement ability of the chain segments in the system, makes it easy to arrange regularly at lower temperatures, and improves the crystallization ability of PLA components. When the content of PEG/CA increased to 15%, *T*_g_ increased, which was the same as the result of DMA. The possible reason was that more PEGCA in the system increased the probability of copolyester reaction with macromolecular chain PLA and increased the chain entanglement with PLA. However, the degree of this non-catalytic esterification reaction is limited. Even if the content of PEG/CA continues to increase, PEGCA still plays a leading role in plasticization, and *T*_g_ shows a downward trend.

**Table 3:** Thermal performance parameters of pure PLA and PLA/PEG/CA blends obtained from DSC secondary heating curve

*W*_PEG/CA_	*T*_g_ (°C)	*T*_c_ (°C)	Δ*H*_c_ (J g^−1^)	*T*_m_ (°C)	Δ*H*_m_ (J g^−1^)	*X*_c_ (%)
0	60.56	98.47	−33.25	167.87	35.19	37.83
5	54.78	93.38	−32.39	167.21	33.15	39.61
10	53.99	82.62	−13.50	168.51	29.28	37.69
15	56.85	78.70	−10.12	167.94	36.15	42.26
20	55.71	—	−10.85	167.82	31.08	41.78

### 3.5. Rheological properties

**Fig. 7:**
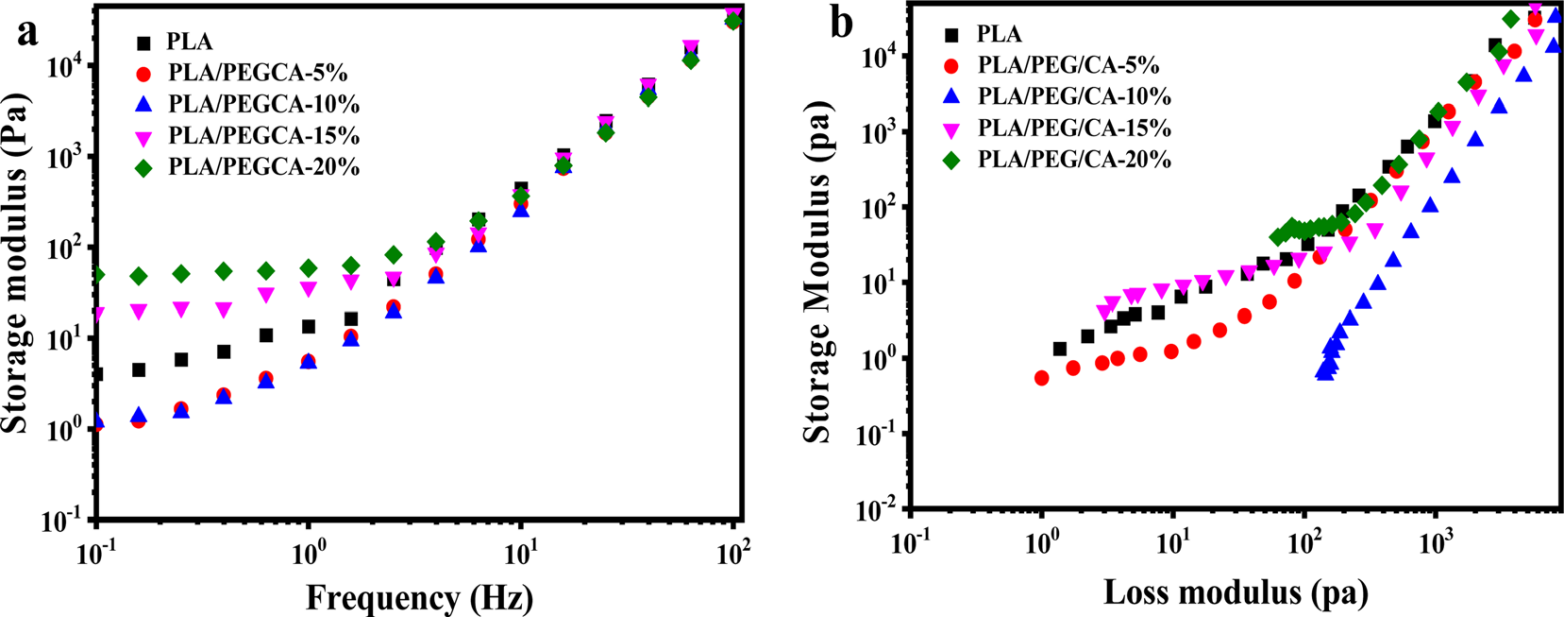
Rheological property of neat PLA and PLA/PEG/CA composites: (a) storage modulus *versus* frequency (b) Han curves of PLA/PEGCA composites at different content.

The relationship curves between storage modulus and shear frequency of PLA/PEG/CA blends with different PEGCA contents are shown in Fig. 7(a). With the increase of frequency, the storage modulus G′ continues to increase. Because at low frequencies, the longer the response time of the blend molecular chain, the more viscous the molecular chain appears, and the smaller the energy storage modulus. With the increase in frequency, the time acting on the molecular chain gradually becomes shorter, and the molecular chain has no time to respond, so it shows the characteristics of rigidity and the storage modulus increases. When the content of PEG/CA was below 10%, the storage modulus of the blend in the low-frequency region was lower than that of pure PLA, indicating that a small amount of PEGCA could not react with PLA to form copolyesters, which mainly played the role of plasticization and increased the mobility of PLA segments. With the continuous increase of PEG/CA content, part of PEGCA reacted with PLA to produce copolyesters, which increased the chain entanglement and the storage modulus of the blends.

Han curve (log *G*′–log G′′) can be used to evaluate the changes in the internal structure of polymers. Fig. 7(b) shows the Han curve of PLA/PEG/CA blends with different PEG/CA contents. When the object of study is blended, the slope at the end of the curve deviates from 2. With the increase of PEG/CA content to 10%, the slope of the Han curve deviated from that of pure PLA, indicating that the blend was only plasticized, and its compatibility was poor; when the content of PEG/CA was 15%, the slope of the Han curve of PLA/PEG/CA was smaller, which was closer to the pure PLA curve, indicating that the compatibility was improved, which also confirmed that the system interface was entangled, and PEGCA reacted with PLA to form a copolyester structure.

### 3.6. Mechanical properties of PLA/PEG/CA blends

**Fig. 8:**
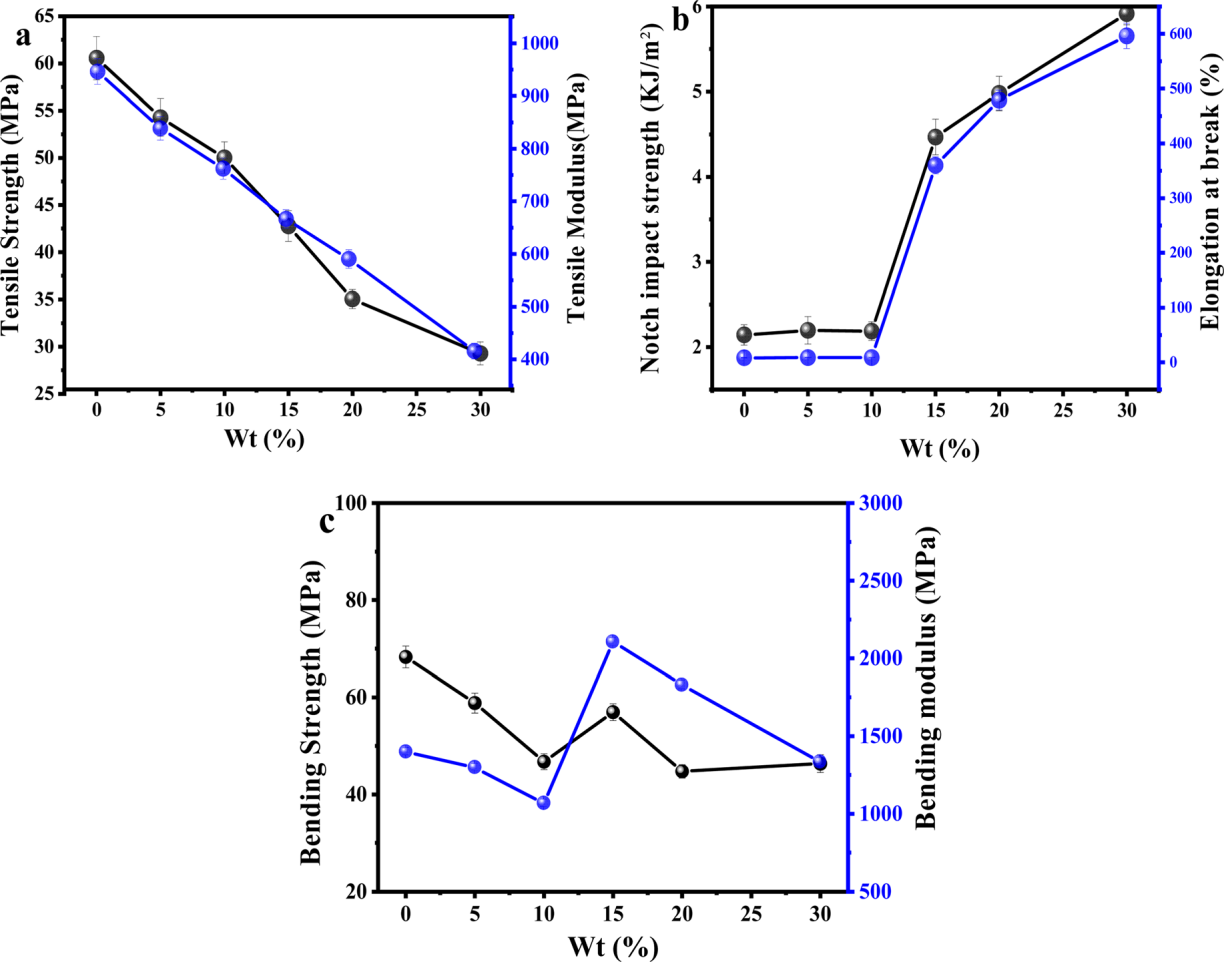
Effect of different dosage ratios of PEG/CA on the mechanical properties of PLA/PEG/CA blends.

The effects of different ratios of PLA/PEG/CA on the mechanical properties of the blends are shown in Fig. 8. Pure PLA is brittle at fracture, with an elongation at fracture of only 9.5%, an impact strength of 2.14 kJ m^−2^, and a tensile strength of 60.56 MPa. When the content of PEG was lower than 15 wt%, the elongation at fracture does not change much, and the tensile strength of the blend varies in the range of 47–59 MPa; when the content of PEG was 15 wt%, the elongation at fracture of the blend is as high as 360.1%, and the tensile strength of the blend was 42.7 MPa. When the content of PEG was 20 wt%, the elongation at the break of the copolymer reached 479.2%, and the elongation at the break was increased by about 50 times. As shown in Fig. 8(b), the impact strength of the copolymers gradually increased with the increase of the mass fraction of PEG/CA, and the notched impact strength of the copolymers reached 4.47 kJ m^−2^ and 5.89 kJ m^−2^ at a PEG content of 15 wt% and 30 wt%, respectively. The impact damage process of the copolymers can be classified into three phases in accordance with the principle, that is the crack initiation phase, interfacial debonding, and crack fracture. With the increase of the PEG/CA content, the more part of interfacial debonding, the more energy is absorbed, so the impact strength increases with the increase of PEG content. The tensile strength of the material decreased to 29.27 MPa with the increase of PEG/CA content, which was in line with the general rule of plasticizer addition. It showed that the reaction between PEGCA and PLA was limited and failed to produce more chain entanglement.

Fig. 8(c) shows the effects of different PEG/CA contents on the flexural strength and flexural modulus of the blend. When the PEG content was below 15 wt%, the plasticizing effect dominates, and the bending strength and modulus of the blend are lower than those of pure PLA. However, when the content of PEG increased to 15 wt%, the increase in bending strength and modulus of the blend may be due to the copolyester of PEGCA-*g*-PLA in the system increasing the chain entanglement nodes with PLA, significantly reducing the material's deformation ability and thereby improving the material's bending modulus. When the PEG content was higher than 15 wt%, maybe due to the low degree of reaction between PEGCA and PLA, the flexible segments of PEGCA in the blend system increased. Therefore, the bending modulus decreased and the material flexibility increased.

### 3.7. Morphology of the blends

**Fig. 9:**
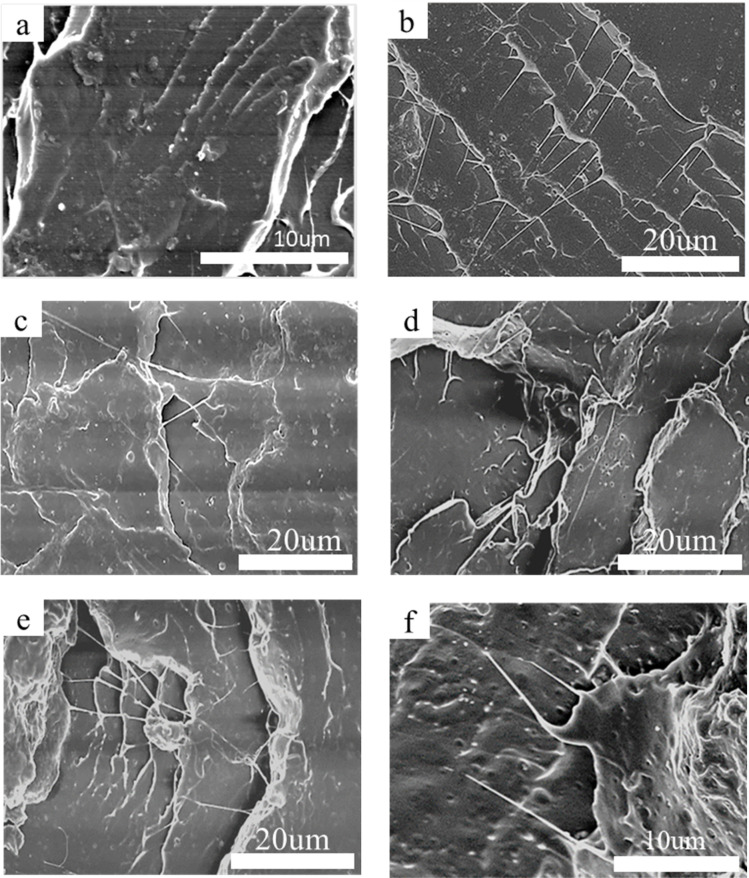
SEM micrographs for impact fractured surface of neat PLA (a), PLA/PEG/CA-5 (b), PLA/PEG/CA-10 (c), PLA/PEG/CA-15 (d), PLA/PEG/CA-20 (e), PLA/PEG/CA-30 (f).

Fig. 9 shows the impact section morphology of pure PLA and PLA/PEG/CA blends with different proportions. It can be seen from Fig. 9(a) that the impact section surface of pure PLA was relatively flat, which also corresponded to its low impact strength and brittle fracture. Compared with PLA, the impact fracture surfaces of PLA/PEG/CA blends were relatively rough, showing ductile fracture and wire drawing phenomenon, which may be due to the role of copolyester formed by partial PEGCA in chain extension compatibility of the blend system.^35,36^ At the same time, the PEGCA copolyester formed *in situ* is an elastic polymer, which acts as the stress concentration region in the PLA matrix to cause high shear yield. It can absorb energy and improve toughness. With the increase of PEG/CA content, there was no obvious “island” structure. More small grooves were observed in Fig. 9(f), indicating that the compatibility between the dispersed phase and the matrix was relatively poor. The reaction between polyester PEGCA and PLA matrix was limited, and more copolyesters could not be formed at the interface; when impacted, the dispersed phase falls off from the matrix and forms small grooves, which affects the plastic deformation of the matrix, absorbs more energy and improves the impact strength.

### 3.8. Possible toughening mechanisms for polyesters

**Fig. 10:**
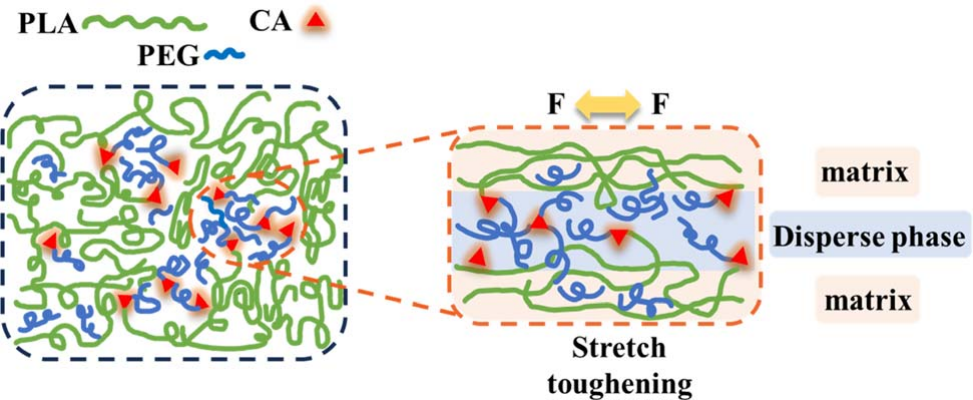
Toughening mechanism of polyester.

Based on the above experimental results, we studied the possible toughening mechanism of the PLA/PEG/CA blend system, as shown in Fig. 10. According to the report, in PLA/PEG blends, the addition of a large amount of PEG will reduce the compatibility between the two, and the PEG segments form droplet-like particles and aggregates embedded in the PLA segments.^35^ After the addition of CA, the carboxyl group of CA preferentially reacted with the hydroxyl group of short chain PEG to form an elastic copolymer ester with a portion of PEGCA as the stress concentration zone, improving the ductility of the material. At the same time, some PEGCA and PLA molecular chains formed copolyesters, improving the compatibility between the matrix PLA and the dispersed phase, increasing entanglement with PLA chain segments, and avoiding a rapid decline in mechanical properties.^27^ However, the results of gel content and mechanical properties showed that the reaction between PEGCA and PLA molecular chain produced by the *in situ* reaction was limited. PEGCA mainly played a plasticizing role in reducing the distance between PLA molecular chains and had limited effect on improving the impact toughness and maintaining the tensile strength of the blends.

## 4. Conclusions

PLA was successfully toughened by PEGCA copolyester, which was formed by *in situ* polymerization of PEG and CA in the PLA matrix during reactive blending. The monomer CA is also used as a chain extender. Its carboxyl group reacts with hydroxyl groups in PLA and PEG to form copolyesters, which increases the interfacial compatibility between PLA matrix and dispersed phase PEG and PEGCA and improves the tensile toughness and notch impact toughness of PLA. When the content of PEG reached 15 wt%, the impact strength of the blend was 4.47 kJ m^−2^, and the maximum elongation at break was 360.1%, which was about 2 times and 44 times higher than that of pure PLA, respectively, and the tensile strength remained at 70%. The toughening mechanism of polyester PEGCA obeys the cavitation mechanism, where the impact triggers the creation of holes between the two phases, and the PLA matrix phase yields in shear. The dispersed PEGCA as the stress concentration point induced shear yield and produced microfibers, which absorbed the impact energy and improved the impact strength. However, the degree of reaction between PEGCA and PLA produced by the *in situ* reaction is insufficient, and its effect on improving the impact toughness and maintaining the tensile strength of the blend is limited, which needs to be further improved in the future.

## Conflicts of interest

There are no conflicts to declare.

## Supplementary Material
